# Mosquito Bite as a Potential Novel Trigger for Acute Generalized Exanthematous Pustulosis (AGEP): A Case Report

**DOI:** 10.7759/cureus.81269

**Published:** 2025-03-27

**Authors:** Matiar Madanchi, Franziska P Wenz, Riccardo Curatolo, Beda Mühleisen, Hazem A Juratli

**Affiliations:** 1 Dermatology, University Hospital Basel, Basel, CHE

**Keywords:** agep, case report, dermatopathology, insect bite, mosquito bite

## Abstract

Acute generalized exanthematous pustulosis (AGEP) is a rare and severe cutaneous adverse reaction characterized by the rapid onset of widespread sterile pustules on an erythematous base. While typically triggered by medications, AGEP has been associated with various agents, including viral infections and insect bites. We present the case of a 51-year-old woman who developed a diffuse pruritic papulopustular and vesicular rash following a mosquito bite. Despite negative tests for viral infections and medication use, histopathological analysis revealed classic features of AGEP. Treatment with systemic corticosteroids led to rapid symptom improvement. This case highlights the importance of considering uncommon triggers, such as mosquito bites, in the evaluation of pustular eruptions. It underscores the need for clinicians to maintain a broad differential diagnosis and consider unusual etiologies when evaluating patients with AGEP-like presentations, particularly in cases lacking typical inciting factors. Further research is necessary to explore the potential association between mosquito bites and AGEP and to elucidate the underlying pathophysiological mechanisms involved.

## Introduction

Acute generalized exanthematous pustulosis (AGEP) is a rapidly evolving generalized pustular eruption that usually presents as an acute form of drug reaction. It is characterized by the rapid development of sterile pustules on an erythematous ground and may have a targetoid appearance. Clinically, the lesions of AGEP are typically non-follicular, occurring within 24-48 hours after trigger exposure [1]. It often presents concurrently with fever, peripheral blood leukocytosis, and general discomfort. Mucosal involvement occurs only in a minority of patients (<20%) and is not characteristic. Among the most common drugs causing AGEP are antibiotics, particularly antibiotics of the β-lactam, cephalosporin, and macrolide group, followed by NSAIDs, antifungal agents, calcium antagonists, proton pump inhibitors, uricostatic drugs, and pembrolizumab. In rare cases, AGEP can occur as a result of a viral infection such as enterovirus, parvovirus, or cytomegalovirus. In rare cases, a bacterial infection has been implicated. There are some reported cases in the literature in which an AGEP was triggered by spider bites [1-3]. In rare cases, no cause can be identified. However, to date, no cases of AGEP following a mosquito bite have been reported in the literature.

## Case presentation

We present a case of a 51-year-old healthy woman who has been experiencing a diffuse pruritic non-follicular papulopustular and vesicular rash on an erythematous ground over her entire body for approximately two weeks.

**Figure 1:**
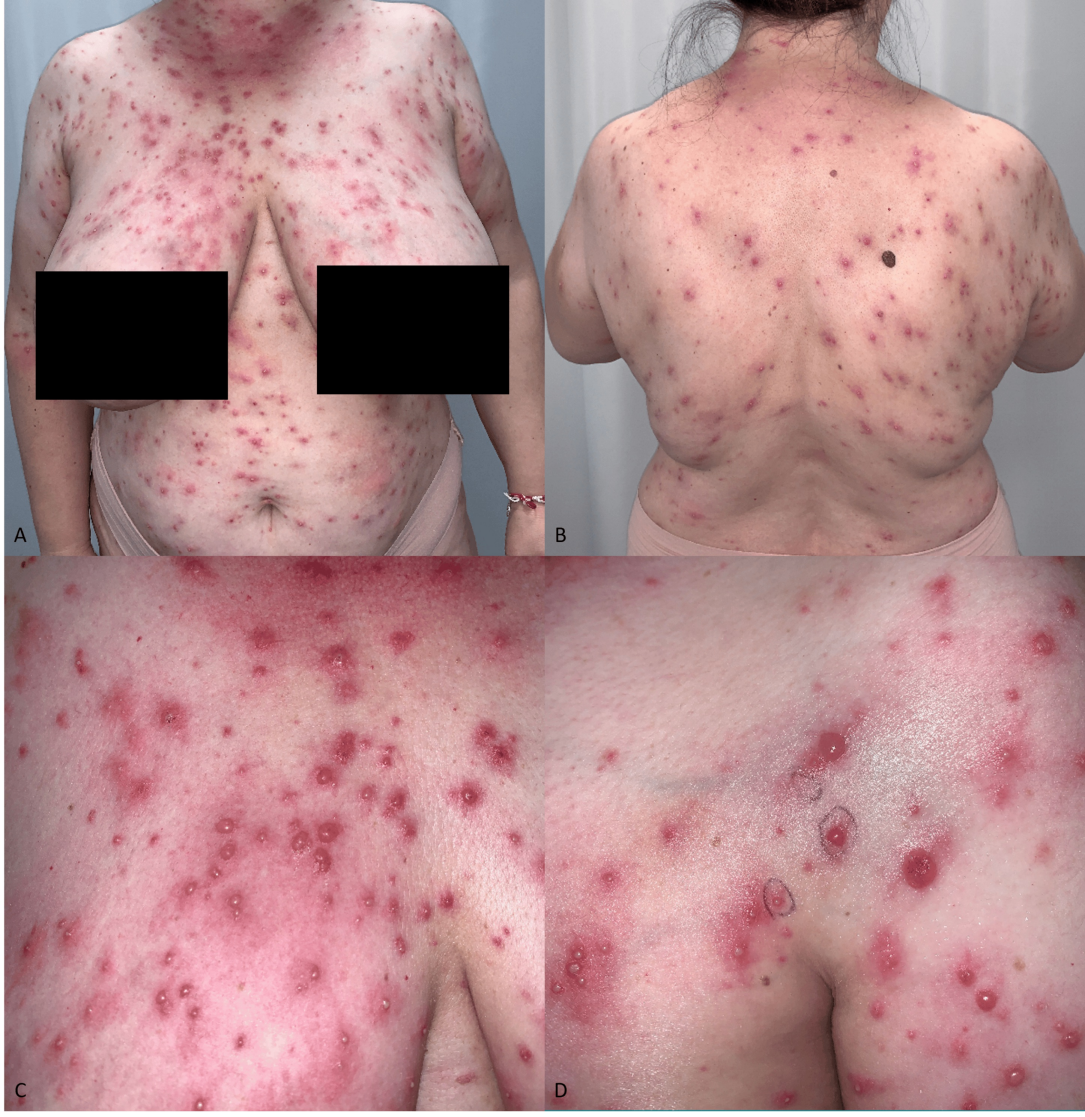
Clinical manifestation A-B: non-follicular papulopustular and vesicular rash on the upper body. C-D: Close-up acquisition of the presence of vesicles, erythematous papules, and pustules on an erythematous ground at the level of the décolleté and the right breast.

The rash was not associated with hair follicles (Figures 1A-1D). The patient reports that it all began when she was out walking in the woods on a summer's day and noticed that a mosquito had bitten her on the right flank. Initially, the bite site was red and very itchy (like a usual skin reaction to an arthropod bite). During her first consultation in our outpatient clinic, the patient showed the images she had taken immediately after the bite on her mobile device. Subsequently, it initially spread in the flexural regions (axillae and groin) and then spread all over her body, except for her face, sparing the mucous membranes. The patient denies recent flu-like symptoms, fever, and recent intake of any new medications.

Table 1 shows different blood tests that were conducted.

**Table 1:** Laboratory findings

Parameter	Value	Normal Range	Unit
White Blood Cells (Leukocytes)	11.1	3.50 to 10.00	G/L
Red Blood Cells (Erythrocytes)	4.62	4.20 to 5.40	T/L
Hemoglobin (Hb)	149	120 to 160	g/L
Hematocrit (HCT)	0.44	0.36 to 0.46	L/L
Mean Corpuscular Volume (MCV)	96	79 to 95	fL
Mean Corpuscular Hemoglobin (MCH)	32.3	27.0 to 33.2	pg
Mean Corpuscular Hemoglobin Concentration (MCHC)	338	320 to 360	g/L
Platelets (Thrombocytes)	256	150 to 450	G/L
Neutrophils (%)	70.2	40.0 to 74.0	%
Neutrophils (Absolute)	7.79	1.30 to 6.70	G/L
Lymphocytes (%)	20.4	19.0 to 48.0	%
Lymphocytes (Absolute)	2.27	0.90 to 3.30	G/L
Monocytes (%)	4.6	3.4 to 9.0	%
Monocytes (Absolute)	0.51	0.12 to 0.62	G/L
Eosinophils (%)	3.9	0.0 to 7.0	%
Eosinophils (Absolute)	0.43	0.00 to 0.30	G/L
Basophils (%)	0.3	0.0 to 1.5	%
Basophils (Absolute)	0.03	0.00 to 0.09	G/L
Sodium (Na)	142	136 to 145	mmol/L
Potassium (K)	3.4	3.4 to 4.5	mmol/L
Chloride (Cl)	103	98 to 107	mmol/L
Calcium (Ca)	2.37	2.10 to 2.65	mmol/L
Creatinine	68	45 to 84	µmol/L
Glomerular Filtration Rate (GFR, CKD-EPI)	89	-	ml/min/1.7
Urea (BUN)	4.6	3.0 to 7.8	mmol/L
Uric Acid	316	173 to 359	µmol/L
Bilirubin	6.8	<15	µmol/L
Aspartate Aminotransferase (ASAT)	16	10 to 50	U/L
Alanine Aminotransferase (ALAT)	15	10 to 50	U/L
Gamma-Glutamyl Transferase (GGT)	22	10 to 71	U/L
Total Protein	74	64 to 83	g/L
Albumin	39	35 to 52	g/L
C-Reactive Protein (CRP)	17.4	<10.0	mg/L
Alkaline Phosphatase (AP)	65	35 to 105	U/L
Lactate Dehydrogenase (LDH)	206	135 to 214	U/L
Pancreatic Amylase	20	13 to 53	U/L
Creatine Kinase (CK)	111	38 to 157	U/L

**Figure 2:**
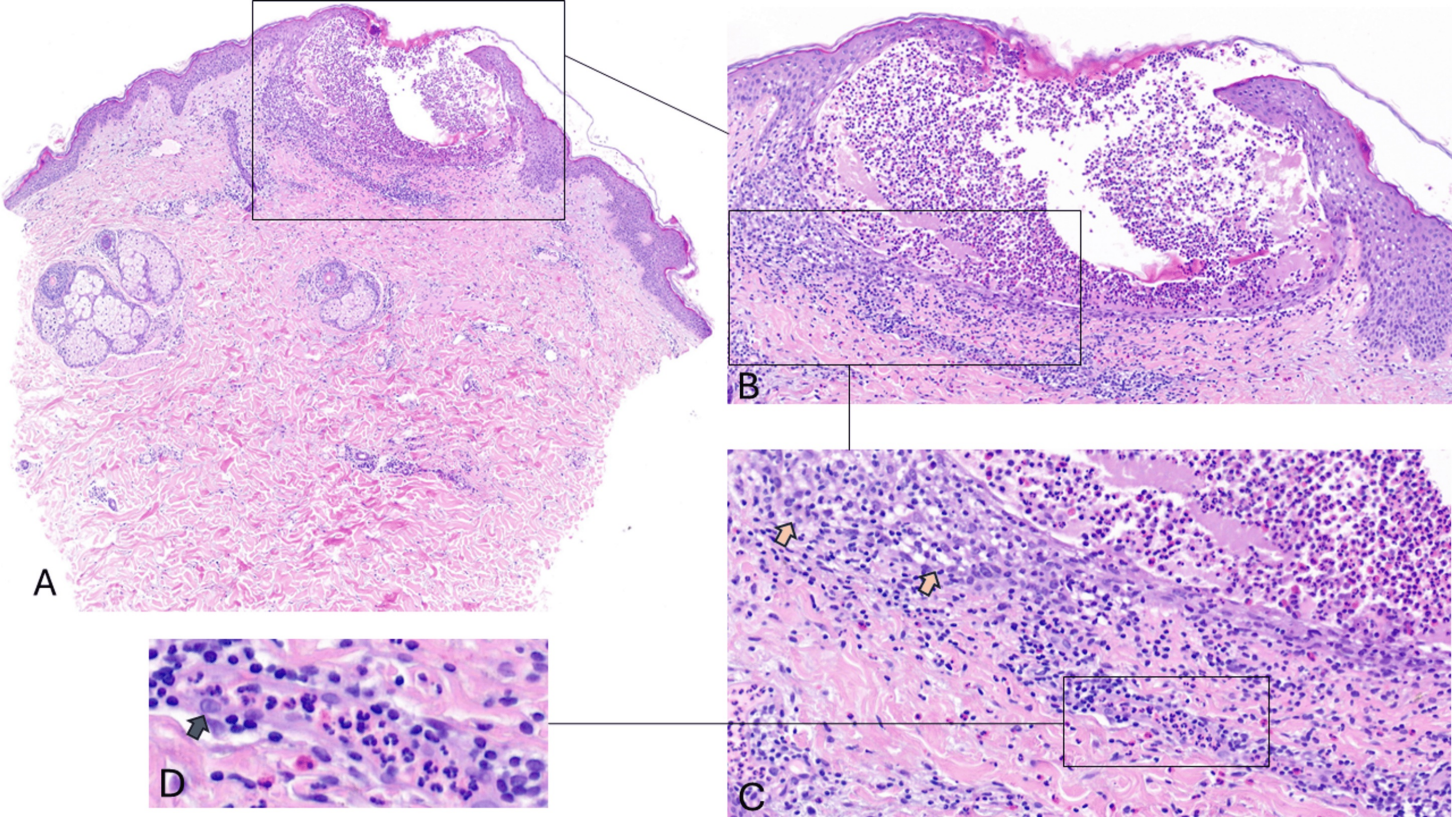
Skin biopsy from a clinical pustule A-D: A: Low-power view of Hematoxylin and Eosin (H&E stain, 12.5x magnification). B: Subcorneal intraepidermal pustule filled with numerous eosinophils and neutrophils and cell debris. (H&E stain, 100x magnification) C: Yellow arrows indicate exocytosis of numerous lymphocytes with numerous vacuoles representing interface dermatitis with scant dyskeratosis. A mixed neutrophilic and eosinophilic superficial dermal infiltrate is shown (H&E stain, 200x magnification). Schematic representation of a dermal vessel from the upper vascular plexus showing intravascular neutrophil granulocytes and endothelial cells swelling (turquoise blue arrow). (H&E stain, 400x magnification)

Tests revealed mild eosinophilia (0.43 G/L; normal range 0-0.3 G/L) (Table 1). A bacterial culture from the pustules was negative. The patient reported that she had chickenpox as a child. Given the presence of multiple pustules and vesicles scattered all over the body except for the face and scalp, a VZV (Varicella-Zoster Virus) infection could not be entirely ruled out. We therefore performed a swab from the vesicles for Varicella-Zoster Virus (VZV) and Herpes Simplex Virus I and II (HSV I/II) which turned out negative. A personal and family history of psoriasis was denied. All nails were clinically inconspicuous. Therefore, we performed a biopsy of a pustule (Figure 2).

**Figure 3:**
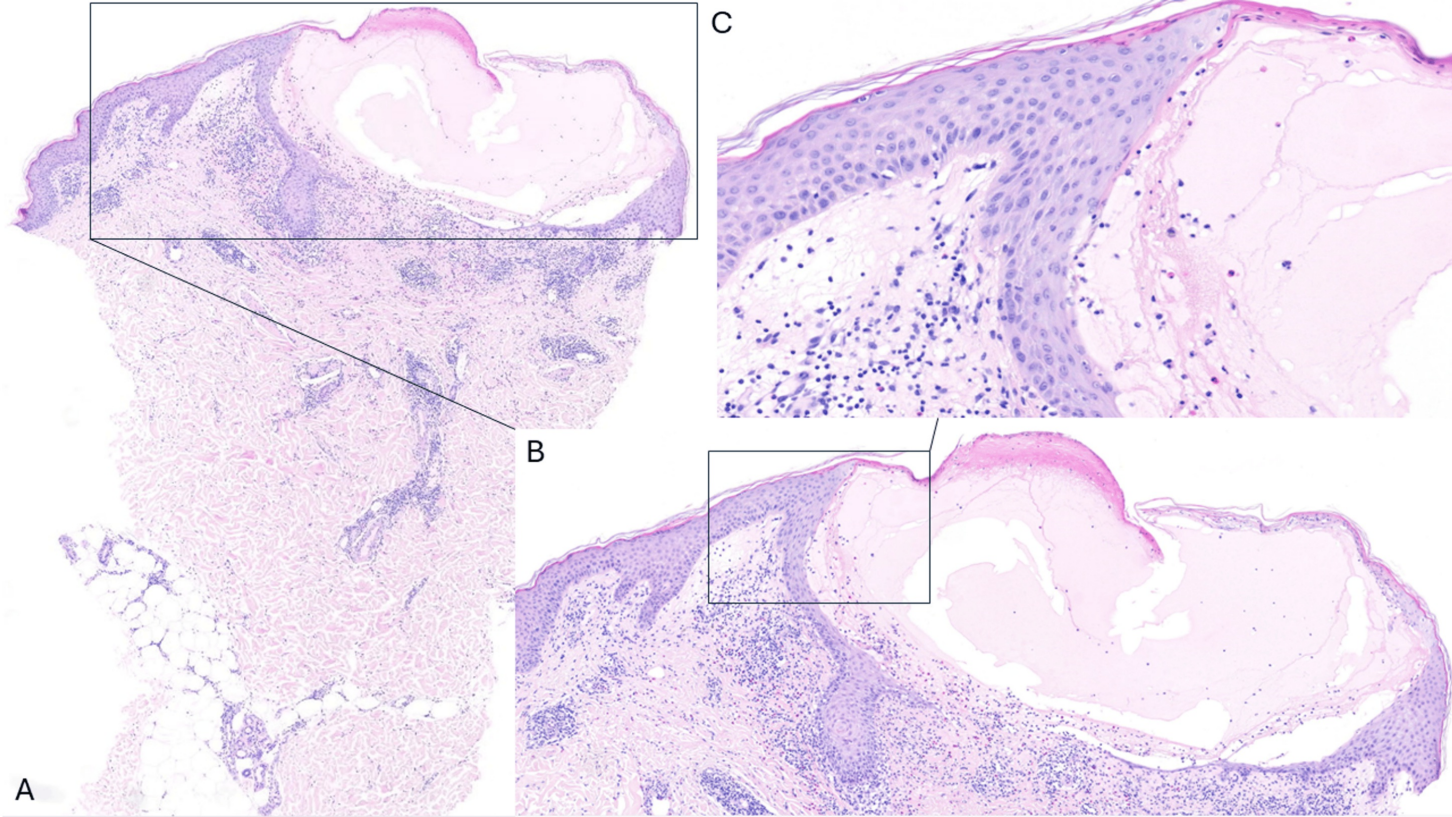
Histopathological Findings of a Clinical Blister A-C: A: Overview at low magnification of hematoxylin and eosin (H&E) staining, 12.5×. B: Presence of a subcorneal intraepidermal large vesicle. In the upper dermis, there is a perivascular and diffuse lymphohistiocytic infiltrate, accompanied by abundant eosinophils and some neutrophils (H&E stain, 100x). C: Larger magnification reveals spongiosis. The dermis appears edematous with a mixed inflammatory infiltrate comprising neutrophils and eosinophils. Notably, there is prominent papillary dermal edema and an absence of tortuous dermal papillary vessels (H&E stain, 200x).

Figures 2A-2D show a subcorneal and intraepidermal pustule filled with numerous eosinophils and neutrophil granulocytes and cell debris. The pustules contained neutrophils in addition to eosinophils. There was exocytosis of numerous lymphocytes with numerous vacuoles representing interface dermatitis with scant dyskeratosis (Figure 2C). A mixed neutrophilic and eosinophilic superficial dermal infiltrate is shown. The dermal vessels from the upper vascular plexus showed intravascular neutrophil granulocytes and endothelial cell swelling (Figure 2D). A biopsy of a vesicle (Figures 3A-3C) showed a subcorneal intraepidermal large vesicle with mild spongiform pustulation at the margins. In the upper dermis, there was a perivascular and diffuse lymphohistiocytic infiltrate, accompanied by abundant eosinophils and some neutrophils. The dermis appeared edematous with a mixed inflammatory infiltrate comprising neutrophils and eosinophils. The papillary dermis was prominently edematous, and there was a heavy mixed inflammatory cell infiltrate in the upper and middle dermis and an absence of tortuous dermal papillary vessels. There were no tissue-resident bacteria detected in the Gram stain. No hyphae were detected in the periodic acid-Schiff (PAS) stain. The mononucleated dermal infiltrate was composed of CD3+ and scattered CD30+ cells. Varicella-Zoster Virus (VZV) immunostaining was also negative. Direct immunofluorescence was negative, ruling out an autoimmune bullous disease.

According to the EuroSCAR group's scoring system (a European project dedicated to severe cutaneous adverse reactions) [4], used in the identification of AGEP cases, the patient received a score of 9, fulfilling the criteria for the diagnosis of AGEP.

From a therapeutic standpoint, we initiated topical steroid treatment with betamethasone dipropionate lotion with limited benefit. We therefore initiated systemic corticosteroid treatment with 0.5 mg/kg prednisolone per day (50 mg) for five days, leading to a rapid improvement in symptoms.

## Discussion

In Table 2, we have summarized the possible causes of AGEP reported. To date, AGEP induced by a mosquito bite has not yet been reported in the literature. Therefore, this could be the first case report of an AGEP induced by a mosquito bite.

**Table 2:** Principal causes of AGEP

PRINCIPAL CAUSES OF AGEP	
Cause	Common Triggers
Drug-induced	Antibiotics (β-lactams and β-lactamase inhibitors, cephalosporins, macrolides, fluoroquinolones, antimalarials)
	Antifungals (terbinafine, miconazole and nystatin)
	Antivirals (acyclovir, favipiravir, remdesivir and ritonavir)
	Calcium channel blockers (diltiazem)
	Others (allopurinol, carbamazepine, glucocorticoids, ibuprofen, metamizole, oxicame, pembrolizumab and paracetamol)
Infections	Chlamydia pneumoniae
	Mycoplasma pneumoniae
	Coccidiomycosis
	SARS-CoV-2
Vaccines	Influenza vaccination
	Spikevax SARS-CoV-2 vaccination
External	Spider bites

Numerous immunological pathways contribute to the pathogenesis of AGEP, resulting in an elevated secretion of interleukin (IL)-8 and subsequent mobilization and survival of neutrophilic granulocytes. Fundamentally, AGEP is believed to arise from a T-cell-mediated delayed-type hypersensitivity reaction triggered by a specific medication or other stimulus. Following exposure to the triggering agent, antigen-presenting cells showcase the antigen on their surface using molecules from the major histocompatibility complex. This triggers the activation of CD4+ and CD8+ T cells that specifically react to the medication, proliferate, and migrate into the dermis and epidermis via perforin/granzyme B and Fas ligands. The subcorneal vesicles are primarily filled with activated CD4+ T cells that release IL-8, which acts as a chemoattractant for neutrophilic granulocytes. In an advanced clinical stage, CD4+ and CD8+ T cells are localized only in the dermis, while the vesicles are filled with neutrophils. This leads to pustule formation in the epithelial region and associated systemic neutrophilia [5-8].

Histologically, AGEP typically exhibits exocytosis with subcorneal collections of neutrophils. Pustules may show spongiotic to unilocular (non-follicular) formation. Focal loss of the stratum granulosum is observed, mostly with orthokeratotic epithelium. Occasionally, eosinophilic granulocytes and extravasated erythrocytes may be present. The pattern involves superficial perivascular and diffuse neutrophilic dermatitis with subcorneal pustule formation [2,9,10].

To facilitate AGEP diagnosis, the EuroSCAR group's scoring system has been developed to provide a precise diagnostic approach. After receiving the histological result, we retrospectively calculated the AGEP EuroSCAR group's scoring system, which showed a total of 9 points, strengthening the diagnostic suspicion.

## Conclusions

In our case, the sole triggering agent was identified as a mosquito bite, and histologically, we could observe many similarities with an AGEP reaction. This reaction could represent a new entity where reactions similar to AGEP, both clinically and histologically, may occur following a mosquito bite.

Thus, it might be a novel entity; however, further studies or additional cases would be needed to confirm our observation/hypothesis.
